# UE Engagement and Bundle Compliance: A Virtual Success Story

**DOI:** 10.1097/pq9.0000000000000487

**Published:** 2021-09-02

**Authors:** Richelle M. Reinhart, Alia Fink, Sopnil Bhattarai, Anit Saha, Katherine Worten, Jessica Cronin, Rahul Shah

**Affiliations:** From the Children’s National Hospital, Washington, D.C.

## Abstract

Children’s Hospitals’ Solutions for Patient Safety (SPS) is a network of over 140 children’s hospitals who share the vision of working together to eliminate serious harm across all pediatric hospitals. The SPS network is built on the fundamental belief that by sharing successes and failures transparently and learning from one another, children’s hospitals can achieve their goals more effectively and quickly than working alone. Each year, SPS hosts National Learning Sessions to which members are invited to submit abstracts describing relevant safety research or improvement work. The following abstracts were among the top submitted for the SPS Spring 2021 National Learning Session.

## Introduction:

COVID-19 forced industries around the world to change processes; this was no different for those working to improve outcomes in healthcare. Many hospitals struggled with the upkeep of hospital-acquired condition auditing and engagement due to competing priorities. The risk of harm does not stop during a pandemic; in fact, it may be increased due to changes in typical care processes. This is particularly true for unplanned extubations (UE), when many hospitals prepared for an increase in patients requiring respiratory care during COVID-19. To address this risk of harm, Children’s National developed a three-pronged approach for virtual engagement and sustainment of the processes necessary to achieve and maintain UE outcomes.

### Goal:

Create a flexible approach to maintaining patient safety during the change to reliance on virtual communication modalities.

### Aim:

Increase the average number of UE audits per month in the intensive care units (ICU) from 34 to 45 by June 30, 2021; increase UE bundle compliance in all ICUs from an average of 81% to >90% by June 30, 2021.

## Methods:

Our three-pronged approach to increasing bundle compliance and audits included: UE leadership meetings, improving the audit process, and ensuring fidelity to bundle elements. Regular meetings were held with ICU leaders using multiple virtual modalities.

## Results:

**Fig. 1.**
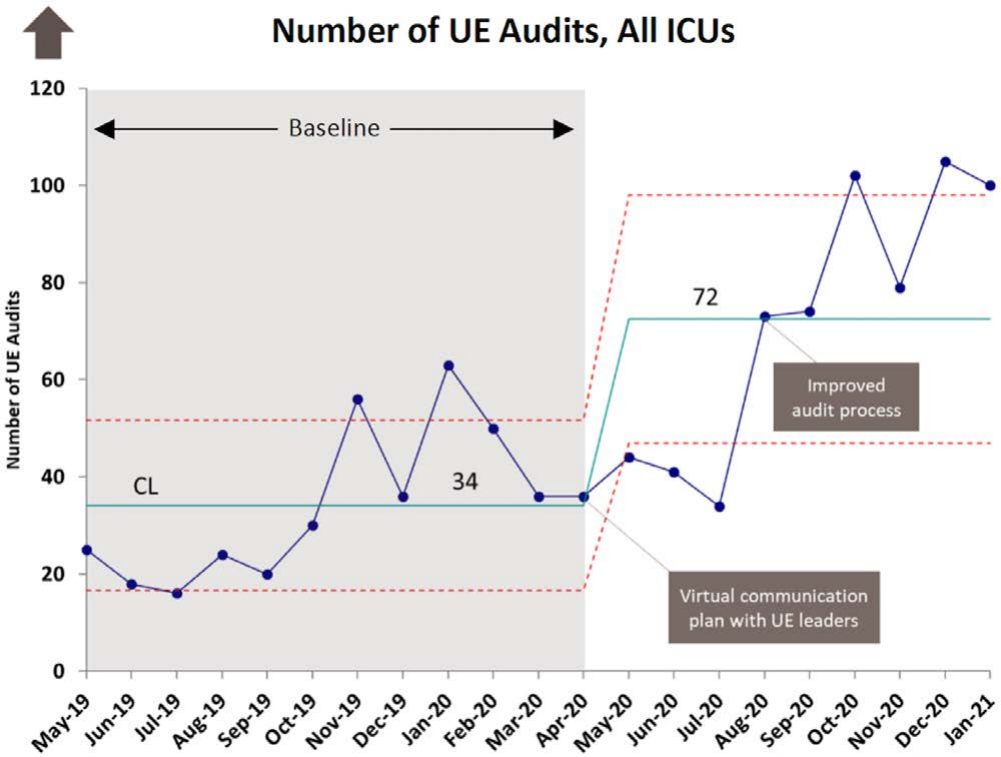
Number of unplanned extubation audits occurring in all ICUs, c-chart.

Qualitatively, we have found that microsystem leaders regularly engage with QI staff and their UE teams using virtual touchpoints and ongoing communication. UE audit numbers increased more than twofold since April 2020, with a change in the mean to 72 audits per month (Fig. 1). Bundle compliance also steadily increased over a similar time period, reaching our goal of > 90% by the end of 2020.

## Conclusions:

In a time of change during a pandemic, increased engagement in hospital-acquired condition work is possible with adapting structure and processes. Our results are reproducible by increasing touchpoints using multiple virtual modalities.

